# Sex Differences in Behavioral Impulsivity in At-Risk and Non-Risk Drinkers

**DOI:** 10.3389/fpsyt.2015.00072

**Published:** 2015-05-13

**Authors:** Jessica Weafer, Jessica De Arcangelis, Harriet de Wit

**Affiliations:** ^1^Department of Psychiatry and Behavioral Neuroscience, University of Chicago, Chicago, IL, USA

**Keywords:** behavioral impulsivity, inhibitory control, impulsive choice, go/no-go, delay discounting, alcohol, AUDIT

## Abstract

**Introduction:**

Mounting evidence from both animal and human studies suggests that females are more vulnerable to drug and alcohol abuse than males. Some of this increased risk may be related to behavioral traits, such as impulsivity. Here, we examined sex differences in two forms of behavioral impulsivity (inhibitory control and impulsive choice) in young men and women, in relation to their level of alcohol consumption and alcohol-related problems (at-risk or non-risk).

**Methods:**

Participants performed a go/no-go task to assess inhibitory control and a measure of delay discounting to assess impulsive choice.

**Results:**

On the measure of inhibitory control, at-risk women committed significantly more inhibitory errors than at-risk men, indicating poorer behavioral control among the women. By contrast, no sex differences were observed between at-risk men and women in delay discounting, or between the male and female non-risk drinkers on any measure.

**Conclusion:**

Heavy drinking women displayed poorer inhibitory control than heavy drinking men. It remains to be determined whether the sex differences in inhibitory control are the result of drinking, or whether they pre-dated the problematic drinking in these individuals.

## Introduction

Alcohol abuse has been traditionally considered a male-oriented problem and as a consequence research on risk factors specific to women has been minimal. However, the sex gap in alcohol consumption and alcohol-related problems is closing rapidly, especially among young adult drinkers (1–4). Specifically, sex differences in frequency and quantity of alcohol consumption, frequency of binge drinking, and prevalence of alcohol abuse and dependence are shrinking, due primarily to increased consumption and prevalence rates in women. In fact, binge drinking rates in women are beginning to surpass those in men in some areas (5). Further, findings from both animal and human studies suggest that females may actually be more vulnerable to drug and alcohol use than males (6–8). Given the increase in alcohol use among women and their increased vulnerability to alcohol-related problems, it is important to identify risk factors for alcohol abuse in women.

One potential risk factor is impulsive behavior. Growing evidence indicates that there are at least two separate components of impulsive behavior: poor inhibitory control (behavioral disinhibition) and impulsive choice (delay discounting), and both forms are strongly implicated in alcohol and drug abuse (9–11). Alcohol-dependent individuals display poor inhibitory control compared to healthy, social drinking controls (12, 13), and poor inhibitory control prospectively predicts the development of alcohol-related problems (14–16). Heavy drinkers also display greater impulsive choice (i.e., steeper discounting of the value of rewards that are delivered after a delay) than social drinking controls (17, 18), and delay discounting prospectively predicts greater alcohol consumption among adolescents over a 2-year period (16).

There is some evidence that healthy men and women differ on measures of impulsive behaviors, although results are mixed and depend on specific tasks administered (19). Regarding inhibitory control, women and girls exhibit poorer inhibition than males on stop signal tasks, which measure the time required to inhibit a response (20, 21). By contrast, men exhibit poorer inhibition on go/no-go tasks, which measure the number of inhibitory failures (22–24). Regarding delay discounting, some studies have found that women discount more than men using hypothetical or chance (based on the role of the die) discounting procedures (25–27), whereas other studies have found greater discounting in men using both hypothetical and chance (based on a lottery) discounting procedures (28, 29). Taken together, sex differences do appear to exist, but the direction of the differences varies across specific domains of impulsive behavior.

To date, only a handful of studies have examined sex differences in impulsive behaviors among problematic drinkers. The interpretation of studies with experienced users is complex, as it is difficult to determine whether any observed behavioral differences pre-dated and contributed to the drinking, or whether the behaviors changed as a result of the drinking. Nevertheless, the findings are informative and useful in designing interventions. Initial evidence shows that heavy, binge drinking women display greater inhibitory deficits compared to both heavy drinking men and light drinkers, on both stop signal and go/no-go tasks (30, 31). By contrast, Bobova et al. (32) found that heavy drinking men discounted a hypothetical monetary reward more than heavy drinking women, although this sex difference was not specific to heavy drinkers. Finally, Yankelevitz et al. (33) examined sex differences in discounting of hypothetical money and hypothetical alcohol in regular drinkers. Although men and women did not differ for either commodity alone, women discounted alcohol more than money, whereas men discounted the two commodities equally. In sum, evidence suggests that poor inhibitory control could be a specific risk factor for heavy, problematic drinking in women, but the current findings regarding sex differences in impulsive choice among drinkers are equivocal.

The current study examined sex differences in both inhibitory control and impulsive choice as a function of drinking status in a community sample of young adult drinkers (*n* = 743). Participants were classified as “at-risk” or “non-risk” drinkers based on their scores on the Alcohol Use Disorders Identification Test [AUDIT; (34)]. The AUDIT is a screening instrument that classifies individuals based on both patterns of alcohol consumption (i.e., frequency and quantity) as well as occurrence of negative alcohol-related consequences. Participants who met the cutoff score of 8 or higher for hazardous drinking were classified as at-risk and those who scored below 8 were considered to be non-risk. Participants performed the go/no-go task to assess inhibitory control and the delay discounting task (DDT) to assess impulsive choice. We hypothesized that overall, at-risk drinkers would be more impulsive on both tasks (i.e., display greater inhibitory failures and steeper delay discounting) compared to non-risk drinkers. Additionally, we hypothesized that among at-risk drinkers, women would display poorer inhibitory control than men. Analyses of sex differences in delay discounting were considered exploratory, given the lack of consistent findings from previous studies.

## Materials and Methods

### Participants

Volunteers were recruited from the community through online and printed advertisements. Inclusion criteria included ages 18–30, at least a high school education, fluency in English, no current or past year diagnosis (including alcohol or substance dependence) on the Diagnostic and Statistical Manual of Mental Disorders, Fourth Edition (35), no lifetime alcohol or substance dependence (other than caffeine or nicotine), and at least some alcohol consumption within the past year. The study was approved by the Institutional Review Board of the University of Chicago, and was carried out in accordance with the Declaration of Helsinki. Participants provided informed consent and were compensated for their time.

### Procedure

These data were obtained in the course of a larger genetic study. Participants attended a 4-h experimental session (morning or afternoon) during which they completed several behavioral tasks and self-report measures in counterbalanced order. Participants were instructed to abstain from alcohol and drugs (other than their usual amounts of caffeine and nicotine) for 24 h before the visit, and breath and urine samples were obtained to verify compliance. After compliance testing, participants completed the tasks and questionnaires reported here.

### Measures

#### Go/No-Go Task

**Figure 1:**
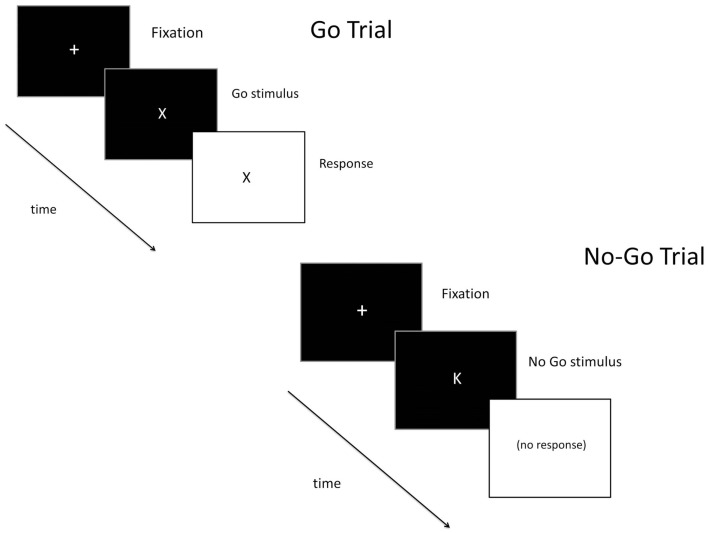
**Schematic of a go (top) and no-go (bottom) trial on the go/no-go task**.

Inhibitory control was assessed using a go/no-go task (Figure 1) that measures the ability to inhibit inappropriate responses. This task has been used extensively in alcohol and drug abuse research, and findings have consistently found that heavy substance use is associated with greater inhibitory errors (9, 36). Go (X) and no-go (K) targets were presented on the computer screen. Participants were told to respond as quickly as possible to go targets but to inhibit their response to the no-go targets. Most (85%) of the trials were go targets, establishing the “go” response as prepotent, and making it more difficult to inhibit when the no-go targets occasionally appeared. The number of inhibitory failures (i.e., failures to inhibit a response to a no-go target) provided the dependent measure of interest. Data were considered invalid if go target accuracy was less than 55% or if there were no successful inhibitions (suggesting a lack of understanding of task instructions).

#### Delay Discounting Task

**Figure 2:**
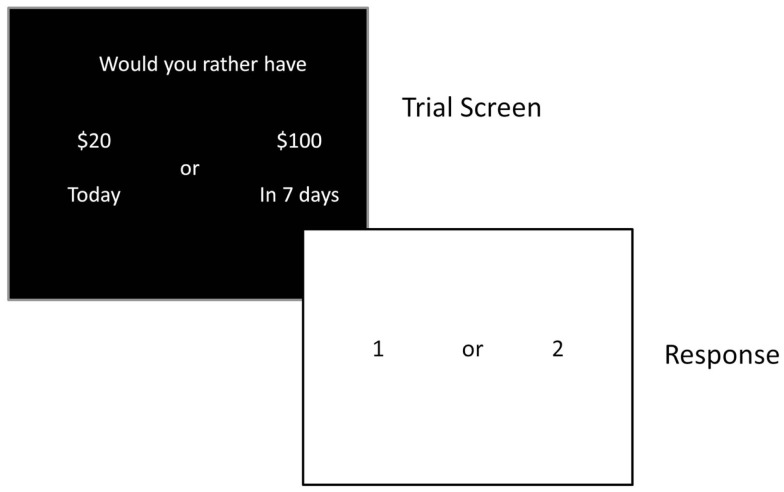
**Schematic of a trial on the delay discounting task**.

Impulsive choice was assessed using a delay discounting task (DDT; Figure 2) that assesses the relative value of immediate versus delayed rewards (37). This task has also been used extensively in drug abuse research, and studies have consistently shown greater discounting of delayed rewards by substance abusers (9, 11, 17). Participants made a series of choices (90 total) between a smaller amount of money (ranging from $10 to $99) delivered immediately, and a larger amount of money ($100) delivered after a delay (i.e., 1, 7, 14, 30, 60, 90, 180, or 365 days). They were told that at the end of the session a random number would be generated and if they guessed the number correctly they would receive the amount of one of their choices. Thus, subjects performed the task knowing that there was a chance they would receive one of their choices. Indifference points were calculated based on the smallest amount of money chosen over the large reward at each delay. Response consistency was calculated at each delay to ensure that participants were performing the task appropriately, and a threshold of 75% consistency was set to indicate adequate effort. The indifference points were plotted to form a discount function, and the area under the curve (AUC) of the discount function provided the dependent measure of impulsive choice (27, 38). A smaller AUC indicates a steeper discounting curve, and therefore greater impulsivity.

#### Alcohol Use Disorder Identification Test

The AUDIT is a 10-item self-report measure that assesses patterns of drinking, dependence, and alcohol-related problems. Scores range from 0 (no alcohol-related problems) to 40 (most severe alcohol-related problems), and a score of 8 or greater is typically indicative of hazardous drinking (34). Accordingly, we classified participants with AUDIT scores less than 8 as “non-risk drinkers” and participants with AUDIT scores of 8 or greater as “at-risk drinkers.”

#### Timeline Follow-Back

Participants completed a retrospective timeline calendar of their alcohol consumption for the past 28 days to assess daily patterns of drinking (39). The measure uses “anchor points” to structure and facilitate participants’ recall of past drinking episodes. For each day, participants estimated the number of standard drinks they consumed. The TLFB provided two measures of drinking habits over the past 28 days: (a) drinking days (total number of days alcohol was consumed) and (b) binge days (number of days in which four or more drinks were consumed for women or five or more drinks were consumed for men). The TLFB was added to the study protocol after the study had begun, and thus data from this measure are only available from a subset of participants (*n* = 457).

### Statistical analysis

The effects of sex and at-risk drinker status on task performance were analyzed by 2 (sex: male vs. female) × 2 (group: at-risk vs. non-risk) between-groups analyses of variance (ANOVA). Significant interactions were followed by *post hoc t*-tests comparing men and women separately in the at-risk and non-risk groups.

## Results

### Sample characteristics

The sample consisted of 743 healthy adults (296 men and 447 women; mean age = 22.9 years, SD = 3.2). Sample characteristics are presented in Table 1. In the sample as a whole, women were slightly younger than men [between-groups *t*-test: *t*(741) = 2.5, *p* = 0.01]. No other sex differences in sample demographics were observed (between-groups *t*-tests: *p*s > 0.64). The racial make-up of the sample was as follows: Asian (*n* = 27), African-American (*n* = 27), Caucasian (*n* = 675), and other (*n* = 14). The majority of participants were Caucasian as these data were collected as part of a larger genetic study.

**Table 1:** **Demographics and drug use characteristics of participants**.

	At-risk drinkers			Non-risk drinkers		
	
	
	Men (*n* = 90)	Women (*n* = 83)	Total (*n* = 173)	Men (*n* = 206)	Women (*n* = 364)	Total (*n* = 570)
Age (mean, SD)	23.3 (3.5)	22.0 (2.8)	22.6 (3.2)	23.3 (3.3)	22.9 (3.1)	23.0 (3.2)
Education in years (mean, SD)	15.3 (2.3)	14.9 (1.9)	15.1 (2.1)	15.4 (2.3)	15.5 (2.0)	15.5 (2.1)
Race (number, %)						
Caucasian	80 (89%)	79 (95%)	159 (92%)	187 (91%)	329 (90%)	516 (91%)
African-American	2 (2%)		2 (1%)	11 (5%)	14 (4%)	25 (4%)
Asian	7 (8%)		7 (4%)	5 (2%)	15 (4%)	20 (4%)
Other	1 (1%)	4 (5%)	5 (3%)	3 (2%)	6 (2%)	9 (1%)
IQ (mean, SD)	119.0 (10.5)	120.3 (10.3)	119.6 (10.4)	119.5 (9.4)	118.7 (9.2)	119.0 (9.3)
Alcohol use measures						
AUDIT (mean, SD)	10.5 (2.5)	10.2 (2.4)	10.3 (2.4)	4.5 (1.7)	4.1 (1.9)	4.2 (1.8)
TLFB^a^ (mean, SD)						
Drinking days/month	13.0 (6.5)	11.0 (5.8)	12.2 (6.2)	8.2 (6.0)	7.4 (5.4)	7.7 (5.6)
Binges/month	4.5 (3.5)	4.4 (3.6)	4.5 (3.5)	1.2 (1.7)	1.3 (1.8)	1.3 (1.7)
Cigarettes/day (mean/SD)	1.1 (2.6)	0.6 (1.3)	0.9 (2.1)	0.6 (1.8)	0.5 (2.2)	0.5 (2.1)
Marijuana (number, %)						
None	31 (34%)	34 (41%)	65 (37%)	125 (61%)	245 (67%)	370 (65%)
Monthly	37 (41%)	37 (45%)	74 (43%)	52 (25%)	94 (26%)	146 (26%)
Weekly	17 (19%)	10 (12%)	27 (16%)	22 (11%)	24 (6.5%)	46 (8%)
Daily	5 (6%)	2 (2%)	7 (4%)	7 (3%)	1 (0.5%)	8 (1%)

*^a^Data gathered from a subset of participants (*n* = 457)*.

### Drinking habits

Slightly less than one quarter of the sample (*n* = 173; 90 men and 83 women) were classified as “at-risk” drinkers (AUDIT scores ≥8) and the remainder (*n* = 570; 206 men and 364 women) were classified as non-risk drinkers (AUDIT scores <8). There were no risk group differences or sex × risk group interactions for any demographic variables (Table 1; *p*s > 0.10). Measures of drinking habits (TLFB and AUDIT) are presented in Table 1 separately for men and women within each group. All alcohol consumption measures were greater in the at-risk compared to the non-risk group (between-groups *t*-tests: *p*s < 0.001). Men and women in the at-risk drinker group did not differ on any alcohol consumption measures (*p*s > 0.05). Among the non-risk drinkers, men had higher AUDIT scores, *t*(568) = 2.9, *p* = 0.003.

### Go/No-Go task

**Figure 3:**
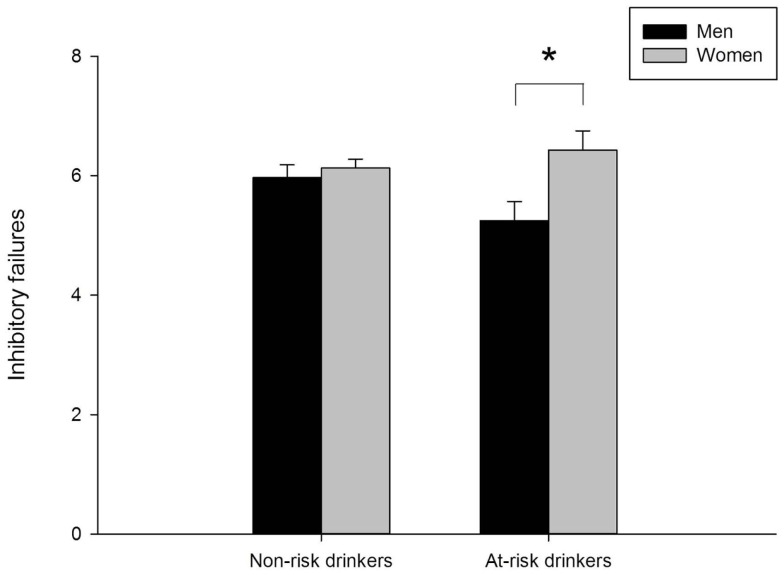
**Mean inhibitory failures on the go/no-go task for men and women in the non-risk (AUDIT scores below 8) and at-risk (AUDIT scores of 8 or above) drinker groups**. In the non-risk group, men (*n* = 190) and women (*n* = 335) did not differ. In the at-risk group, women (*n* = 75) committed significantly more inhibitory failures than men (*n* = 79), *p* = 0.01. Capped vertical lines represent standard error of the mean (SEM).

Valid go/no-go data were obtained for 679 participants (22 participants were missing data and 42 participants had invalid data). Figure 3 presents mean inhibitory failures separately for men and women in the at-risk and non-risk drinker groups. The figure shows that overall women committed more inhibitory failures than men, as evidenced by a main effect of sex, *F*(1, 675) = 6.53, *p* = 0.011. Moreover, the figure shows that the sex difference was more pronounced in the at-risk drinker group compared to the non-risk group. This was confirmed by a significant sex × group interaction, *F*(1, 675) = 3.88, *p* = 0.049. Follow-up between-groups *t*-tests showed significantly more inhibitory failures in women than men in the at-risk group, *t*(152) = 2.58, *p* = 0.011, but no difference in women and men in the non-risk group, *t*(523) = 0.60, *p* = 0.55. No significant differences were observed between risk groups among men or women (*t*s < 1.85, *p*s > 0.05).

### Delay discounting task

**Figure 4:**
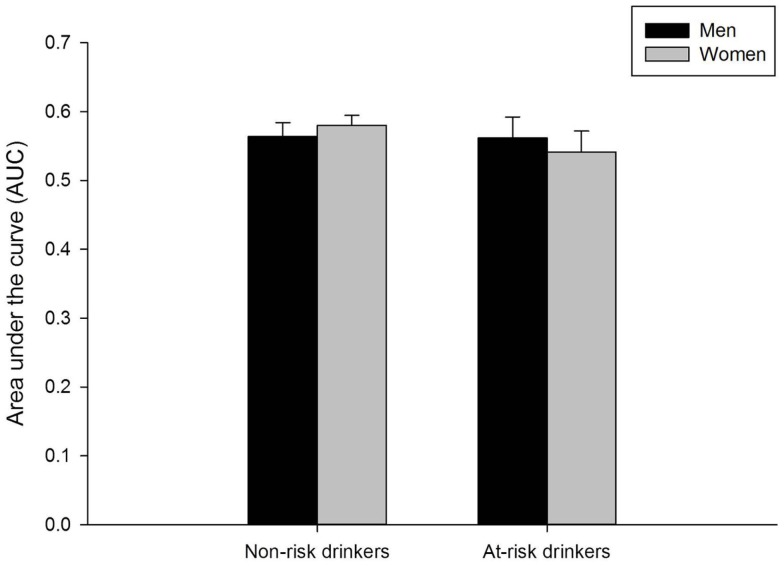
**Mean area under the curve on the delay discounting task for men and women in the non-risk (AUDIT scores below 8) and at-risk (AUDIT scores of 8 or above) drinker groups**. No sex differences were observed in either group. Capped vertical lines represent standard error of the mean (SEM).

Valid delay discounting data were obtained for 734 participants (6 participants were missing data and 3 participants had invalid data). Figure 4 presents mean AUC of the discounting curve separately for men and women in the at-risk and non-risk drinker groups. Neither men and women nor risk groups differed on this measure (*p*s > 0.40).

### Associations between task performance and demographics

Performance on the go/no-go task was not related to delay discounting in the sample as a whole (*r* = 0.01, *p* = 0.73) or when analyzed individually by sex (men: *r* = −0.01, *p* = 0.84; women: *r* = 0.03, *p* = 0.54). Inhibitory failures on the go/no-go task were negatively correlated with age (*r* = −0.20, *p* < 0.001) and education (*r* = −0.08, *p* = 0.04). AUC of the discounting curve was positively correlated with education (*r* = 0.12, *p* = 0.001) and IQ (*r* = 0.22, *p* < 0.001). As greater AUC indicates less discounting, these correlations indicate that greater impulsive choice is associated with lower IQ and less education. Sex differences in both go/no-go task performance and delay discounting were re-analyzed controlling for each of these demographic variables, and the results remained unchanged.

## Discussion

This study examined sex differences in behavioral impulsivity (i.e., poor inhibitory control and impulsive choice) in at-risk and non-risk drinkers. Risk status was determined by scores on the AUDIT, a self-report measure that assesses frequency and quantity of alcohol consumption and alcohol-related problems. As hypothesized, at-risk women displayed poorer inhibitory control than at-risk men, but no sex differences were observed in the non-risk drinkers. On impulsive choice, no differences were observed in either men vs. women or by risk group.

Our findings of sex differences in inhibitory control are largely consistent with previous reports. Specifically, other studies have shown that heavy drinking women exhibit greater inhibitory deficits than heavy drinking men and light drinkers (30, 31). Further, in previous studies of women only, heavy drinking women show greater inhibitory deficits than light drinking women (40, 41). Although statistically significant differences were not observed between the at-risk and non-risk women in the current study, the direction of findings are in line with these reports and provide further support for inhibitory deficits among hazardous female drinkers. Regarding impulsive choice, the current findings are not consistent with one prior report of greater discounting among men compared to women in a sample of both alcohol-dependent individuals and controls (32). As no participants met dependence criteria in the current study, it could be that sex differences in delay discounting are more pronounced among individuals with alcohol use disorders. Taken together, these findings suggest that problematic alcohol consumption in women is strongly linked to poor inhibitory control, but not delay discounting.

Given mounting evidence of a link between disinhibition and heavy drinking in women, it is important to determine the causal direction of this association, as inhibitory deficits could be either a cause or consequence, or both, of heavy drinking. Evidence that sex-specific biological factors contribute to poor inhibition in non-alcohol abusing women would suggest that inhibitory deficits precede the onset of heavy drinking. For example, sex differences in circulating levels of gonadal hormones, including estradiol (E2), could influence inhibitory control. Indeed, Colzato et al. (20) showed that women exhibit poorer inhibition than men only when E2 levels are high, and that poorer inhibition is correlated with higher salivary measures of E2. Additionally, sex differences could exist in activation of neural circuitry underlying inhibitory control. Initial neuroimaging studies have reported that this circuitry is less strongly activated during response inhibition in women compared to men (42–45), although there are also reports of less activation in men (46), or complex differential patterns of activation in men and women (47). In sum, there is preliminary evidence of biologically based mechanisms underlying sex differences in inhibitory control, suggesting that poor inhibition may precede, and be a risk factor for, excessive and problematic alcohol use in women.

Alternately, evidence that women are more sensitive to the neurotoxic effects of alcohol would suggest that observed inhibitory deficits in women are a consequence of heavy drinking. Although findings are mixed, there is some evidence of greater adverse effects of alcohol on brain structure in adult female compared to male alcoholics [for review, see Ref. (48)]. Further, in a sample of adolescents, Squeglia et al. (49) observed thicker cortices (indicative of less synaptic pruning) in frontal regions in binge drinking females compared to controls, as well as an association between thicker cortices and poorer inhibition in females. This group also observed decreased brain activation in female binge drinkers compared to controls during performance of a spatial working memory task, and decreased activation was associated with poorer task performance (50). Although no studies to date have examined neural activation underlying poor inhibitory control in heavy drinking female adolescents or adults, there is evidence to suggest that females may be more sensitive to the adverse effects of alcohol on inhibition-related brain structure and function.

There are several limitations to this study. First, we did not specifically recruit for heavy drinkers and excluded any potential volunteers with a history of alcohol dependence. As such, non-risk drinkers were over-represented in this sample, and this may have contributed in part to our failure to replicate well-established findings showing greater impulsive behavior in hazardous drinkers. Indeed, meta-analyses of impulsive behavior (poor inhibitory control and greater impulsive choice) report the most pronounced effects when alcohol dependent individuals are compared to healthy controls, and much weaker effects for non-dependent drinkers compared to controls (13, 17). It will be important for future studies to examine sex difference in impulsive behaviors within alcohol dependent populations, while taking into account other psychiatric symptoms that could influence sex differences, such as anhedonia (51). An additional limitation of the sample is the over-representation of women. It is important to note, however, that numbers of men and women were balanced within the at-risk drinker group. A third limitation is the lack of assessment of sex hormones. Circulating levels of gonadal hormones influence both inhibitory control (20) and impulsive choice (29, 52), and it is crucial that future studies examining sex differences in impulsive behavior account for the role of hormones in any observed differences.

In sum, this study adds to the existing literature suggesting that poor inhibitory control is strongly linked to problematic alcohol consumption in women. Future longitudinal research is needed to determine whether poor inhibitory control is a cause, or consequence, or both of heavy drinking in women. A better understanding of this association will allow for the development of sex-specific prevention and treatment efforts for alcohol abuse, with a focus on the role of poor inhibitory control.

## Author Contributions

JW and JDA oversaw data acquisition and management, conducted the data analyses, and conducted the literature review and co-wrote the first draft of the paper. JW, JDA, and HdW contributed to and approved the final version of the paper.

## Conflict of Interest Statement

The authors declare that the research was conducted in the absence of any commercial or financial relationships that could be construed as a potential conflict of interest.
